# A pre–post intervention study of pulmonary rehabilitation for adults with post-tuberculosis lung disease in Uganda

**DOI:** 10.2147/COPD.S146659

**Published:** 2017-12-11

**Authors:** Rupert Jones, Bruce J Kirenga, Wincelsas Katagira, Sally J Singh, Jill Pooler, Alphonse Okwera, Richard Kasiita, Doyo G Enki, Siobhan Creanor, Andy Barton

**Affiliations:** 1 Population Studies and Clinical Trials, Peninsula Schools of Medicine and Dentistry, Plymouth University, Plymouth, UK; 2 Population Studies and Clinical Trials, Makerere Lung Institute, Makerere University College of Health Sciences, Mulago Hospital, Kampala, Uganda; 3 Cardio-Respiratory Directorate, University Hospitals of Leicester NHS Trust, Leicester; 4 Population Studies and Clinical Trials, Peninsula Schools of Medicine and Dentistry, Plymouth University, Plymouth, UK; 5 Department of Physiotherapy, Mulago Hospital, Kampala, Uganda; 6 Medical Statistics, Peninsula Schools of Medicine and Dentistry, Plymouth University, Plymouth, UK

**Keywords:** tuberculosis, exercise training, self-management, nonpharmacological intervention

## Abstract

**Setting:**

The study was conducted at Mulago Hospital, Kampala, Uganda.

**Objective:**

As chronic respiratory disease (CRD) is a huge, growing burden in Africa, with few available treatments, we aimed to design and evaluate a culturally appropriate pulmonary rehabilitation (PR) program in Uganda for people with post-tuberculosis lung disorder (p-TBLD).

**Design:**

In a pre–post intervention study, a 6-week, twice-weekly PR program was designed for people with p-TBLD. Outcome measures included recruitment, retention, the Clinical COPD Questionnaire (CCQ), tests of exercise capacity, and biometrics. Given this was a developmental study, no formal statistical significance testing was undertaken.

**Results:**

In all, 34 participants started PR and 29 (85%) completed all data collection. The mean age of the 29 participants was 45 years, and 52% were female. The mean (95% confidence interval) CCQ score at baseline was 1.8 (1.5, 2.0), at the end of PR was 1.0 (0.8, 1.2), and at 6 weeks after the end of PR was 0.8 (0.7, 1.0). The Incremental Shuttle Walking Test (ISWT) was 299 m (268.5, 329.4) at baseline, 377 (339.6, 413.8) at the end of PR, and 374 (334.2, 413.5) at 6 weeks after the end of PR. Improvements were seen in measures of chest pain; 13/29 (45%) participants reported chest pain at baseline but only 7/29 (24%) at the end of PR, and in those with persistent pain, the mean pain scores decreased. Mild hemoptysis was reported in 4/29 (17%) participants at baseline and in 2/29 (7%) at the end of PR.

**Conclusion:**

PR for people with p-TBLD in Uganda was feasible and associated with clinically important improvements in quality of life, exercise capacity, and respiratory outcomes. PR uses local resources, requires little investment, and offers a new, sustainable therapy for p-TBLD in resource-limited settings. With the rising global burden of CRD, further studies are needed to assess the value of PR in p-TBLD and other prevalent forms of CRD.

## Introduction

The World Health Organization considers the control and management of noncommunicable diseases (NCDs) a top priority – NCDs cause more deaths than all other causes combined and are projected to increase from 38 million worldwide in 2012 to 52 million by 2030.^1^ Lung diseases are preeminent, and COPD is now the third leading cause of death globally and the ninth highest cause of disability.^1^ The burgeoning prevalence of chronic respiratory disease (CRD) is fueled by an aging population, the combination of respiratory infections such as tuberculosis (TB) with human immunodeficiency virus (HIV), tobacco smoking, household air pollution, and nutritional impairment.^2^–^4^

One major cause of CRD is post-tuberculosis lung disorder (p-TBLD). In a study in the USA, the long-term sequelae of pulmonary tuberculosis (PTB) dwarf the costs and disease burden of acute infection.^5^,^6^ In Africa, the problem may be worse; permanent extensive lung damage after PTB is common and is associated with delayed diagnosis, multidrug resistance, and nonadherence to treatment.^7^ In addition, air pollution (both indoor and outdoor) and tobacco smoking are pervasive problems that add to damage to lung parenchyma from repeated infections.^2^ An informal notes review of sequential patients attending respiratory clinics in Mulago Hospital, a tertiary referral center in Uganda, showed that 30% of those attending the TB clinic had p-TBLD and 12% of those attending general chest clinics had p-TBLD as the main presenting problem. The range of clinical features included dyspnea, chest pain, and hemoptysis, similar to patients seen in Rwanda.^8^ Currently, there is little or no effective treatment for people with p-TBLD in Africa.

People with CRD are prone to develop musculoskeletal dysfunction related to physical inactivity and systemic inflammation, compounded by impaired nutrition.^9^ Such people enter a vicious circle with falling body weight, progressive morbidity, and increased mortality, accelerated by the accompanying anxiety, depression, and social isolation. Symptoms such as breathlessness, fatigue that comes with deconditioning, demotivation, and psychosocial isolation have been shown previously to be amenable to pulmonary rehabilitation (PR).^10^,^11^

PR is a program of individually prescribed and supervised exercise, education, and self-management activities. There is strong evidence that PR improves health status, exercise capacity, and social functioning, and PR is recommended in international guidelines^10^,^11^ with supporting information on delivery.^12^ PR may be delivered using existing local staff such as nurses, doctors, physiotherapists, and clinic staff and allows patients to help themselves and to help each other. As there is no need for major capital outlay or equipment, PR has the potential to be a sustainable and scalable intervention in resource-limited settings. However, the potential for PR to improve CRDs, such as COPD and p-TBLD, in low- to middle-income countries (LMICs) has not been properly tested. A literature review found no evidence of PR being used in sub-Saharan Africa to treat p-TBLD or indeed any CRD, apart from an observational study in 2001 in 38 people with asthma and COPD,^13^ and for patients undergoing treatment for active pulmonary TB, a pilot trial of PR in South Africa showed some promise.^14^ Globally, there is little evidence of PR being used to treat p-TBLD;^15^ there were two observational studies of PR from Japan in elderly people with p-TBLD, one with 32 participants and one with 37 participants, and more recently one from Colombia including 11 p-TBLD patients.^16^–^18^

In 2014, we conducted a successful feasibility study of running PR for people with p-TBLD in Kampala. We designed and established a process of recruitment, assessment, intervention, and follow-up and trained local staff to deliver the program. In the 18 participants completing the feasibility study, clinically important improvements were seen in the quality of life, exercise capacity, and biometrics. An unexpected reduction in the number of participants reporting chest pain and hemoptysis was also noted.

The aim of our current study was to design a culturally appropriate program for people with p-TBLD and examine impact of PR on quality of life, exercise capacity, and other impacts to inform the development of a larger trial.

## Patients and methods

### Study design

This study was a pre- and post interventional cohort study.

### Participants

Patients attending the respiratory outpatients department in Mulago Hospital were screened, and those potentially suitable were invited to take part in more detailed assessments.

The inclusion criteria were previous treatment for pulmonary TB and breathlessness reaching Medical Research Council (MRC) dyspnea scale grade 2 or higher. We excluded those with sputum positive testing for TB (GeneXpert), unstable cardiovascular disease, and locomotor difficulties that precluded exercise; those who were unwilling or unable to attend a PR program; and those unable to provide informed consent.

### Assessments

The assessments comprised a medical review, physical examination, and review of relevant investigations (eg, chest X-ray, spirometry, sputum for TB using GeneXpert, HIV test). Written informed consent was obtained, and in the case of participants aged <18 years, the parents provided written informed consent. Patients meeting inclusion criteria underwent further rehabilitation assessments including demographics, questionnaires (Karnofsky scale,^19^ MRC dyspnea scale,^20^ Clinical COPD Questionnaire [CCQ],^21^ and Patient Health Questionnaire-9 [PHQ-9]),^22^ biometrics, and tests of exercise capacity (Incremental Shuttle Walking Test [ISWT]^23^ and Five Times Sit-to-Stand test).^24^ We also captured details of symptoms, including a rating scale of chest pains based on the Brief Pain Inventory. To ensure that patients were suitable, a final review of all patients being assessed was undertaken by a physiotherapist and physician, with particular emphasis on those with a Karnofsky score^19^ of 0–30 (too sick) or >80 (not disabled) or an ISWT distance of >600 m at the baseline assessment.

An extensive range of qualitative assessments were undertaken and will be reported separately.

### PR program

Based on conventional PR models, the program was conducted twice weekly for 6 weeks with groups of 10–13 participants and consisted of exercise and education. The day-to-day program was overseen by a senior physiotherapist in collaboration with a specialist nurse, counselor, and doctor. The sessions were conducted in the physiotherapy department, which included an outdoor but shaded walking area. The program lasted ~2 hours; drinks and light food were provided.

The exercise program followed international guidance for PR.^10^,^12^ The main focus of the training program was aerobic lower limb exercise. This was predominately walking based, and the prescription was based upon initial performance on the ISWT. The intensity of the program was set at 80% of peak performance on the ISWT. The exercise regime was individually prescribed, monitored, and increased as the program progressed under the supervision of the physiotherapist (RK). The aim was to complete 30 minutes of walking at the set speed five times a week. At each session, participants also completed resistance training for the upper and lower limbs. The stations included sit-to-stand, step-ups, biceps curls, and upright rowing with weights. The weights were increased as each participant achieved three sets of eight to ten repetitions of each exercise. Exercise was followed by group warm down and education. Participants received instructions on a home exercise regime (both aerobic and resistance), and they kept a diary of the exercises undertaken at home. After completion of the program, participants were encouraged to continue with the exercises in the long term. The program deployed little specialist equipment and was improvised for the resistance training, particularly at home.

The education component included information on pulmonary TB specifically and CRD generally and covered the causes and effects of CRD, self-management, avoiding adverse exposures, exercise, and diet. The PR program operated to the British Thoracic Society PR Quality Standards.^12^ Participants were provided with an information sheet on the PR program and details of exercises to do at home during and after the program. (A video about the program is available at https://www.plymouth.ac.uk/research/primarycare/fresh-air.)

Patients and hospital staff were involved in the design to ensure that the venue, timing, and access were addressed. The PR program was strongly based on Western evidence-based models, and relatively little cultural adaptation was wanted or needed. For example, using local music or dance to make the program more interesting and appropriate was rejected by both participants and staff. The adaptations to make the program relevant to the health care setting were not difficult as first, it was delivered by local clinicians in the National Referral Hospital and second, the UK model that the service was developed from was used with minimal resources.

### Statistical methods

Outcomes are summarized descriptively, with estimates accompanied by 95% confidence intervals where appropriate, and presented in tables and graphs. Given this was a development study, no statistical significance testing was undertaken, as the study was not designed a priori to have sufficient statistical power to detect differences in clinical outcomes; however, where appropriate, the average changes in clinical outcomes are discussed in relation to published minimal clinically important differences (MCIDs).

### Ethical approval

Ethical approval was obtained from the Mulago Hospital Research and Ethics Committee MREC number 440 (28.10.2013).

### Trial registration

The study was registered at the International Standard Registered Clinical/soCial sTudy Number (ISRCTN) registry (http://www.isrctn.com) under number ISRCTN14312425.

## Results

In total, 113 patients attending the general respiratory outpatients department of Mulago Hospital were assessed using a screening form for potential p-TBLD, of whom 56 met the screening criteria of MRC dyspnea scale 2 or above and previous TB treatment, and were further assessed for suitability for this study. Of the 56 patients, 36 met all the entry criteria, 34 started PR in three groups, and 29 completed the program and all data collection. Of the five participants failing to complete the 6-week program, three did not reappear for unknown reasons, one died, and one was diagnosed with recurrent PTB infection and admitted for treatment.

The baseline characteristics of the 29 participants who completed the program are shown in Table 1. Just over half of the participants (51.7%) were female, and the mean age was 45 years, range 17–69 years. Spirometry showed 14/29 (48.2%) participants had obstruction, 10/29 (34.4%) had restriction, and 5/29 (17.2%) had normal results at baseline. Reversibility testing was performed on those with forced expiratory volume (FEV):forced vital capacity (FVC) ratio <0.7: of the 12 participants undertaking this, only two showed reversibility of ≥12% and >200 mL change in forced expiratory volume in 1 second (FEV_1_). Further spirometric data are shown in Table 2. Before exercise, 7/29 (24.1%) participants had resting oxygen saturations of <93% versus 11/29 (37.9%) participants after completing the maximal exertion (the ISWT).

Health status results showed that after completing PR, the mean improvement in the CCQ was 0.95 (Table 3) and the total and domain score improvements all exceeded the MCID of −0.4.^25^ The mean improvement in the ISWT was 90 m (MCID 48 m), and the improvement in Sit-to-Stand test time was 2.5 seconds, also beyond the MCID (MCID 2.3 seconds).^26^ At the individual level, 17/29 (59%) participants showed improvements in ISWT and 18/29 (61%) participants in the Sit-to-Stand test above the respective MCIDs. The mean Karnofsky scale score, which rates people’s status between 0 (dead) and 100 (normal health), improved by 13.8%. Depression scores on the PHQ-9 were very low at baseline and almost zero after the program; the proportion of participants with a score of ≥5 was 7/29 (24.1%) at baseline and 0/29 at the end of PR and after 6 weeks.

Biometrics also showed that from a low baseline, there were modest increases in average body mass index (BMI) and mid-upper arm circumference (Table 3). Three out of 14 participants whose BMI was <20 at baseline had BMI scores in the normal range (20–25) immediately and 6 weeks after rehabilitation.

**Figure 1:**
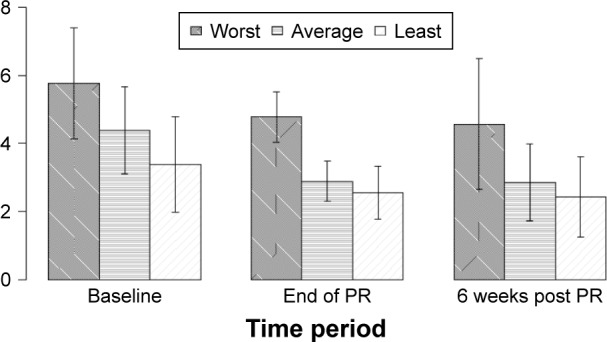
Mean chest pain severity scores before, after and six weeks after PR (n=29). **Abbreviation:** PR, pulmonary rehabilitation.

Measures of participants’ symptoms showed a reduction in the proportion with chest pain and hemoptysis but not with cough (Table 4). Chest pain was reported in 13/29 (45%) participants prior to PR but only in 7/29 (24%) participants after 6 weeks; thus, the pain was abolished in nearly half the people completing PR. Group mean chest pain scores fell (Figure 1), and even in the participants who still reported pain at the study end, the pain severity scores fell in 7/13 (54%) participants. The nature of the pain was consistent in participant reports; it was described as sharp, severe, and worse with lying down. For many participants, pain was as big a problem as breathlessness, and its reduction was reported as a great benefit. Mild hemoptysis was reported in 4/29 (17%) participants at baseline and in 2/29 (7%) participants at the end of PR and after 6 weeks.

## Discussion

In this study, we aimed to examine the impact of a PR program in Uganda for people with p-TBLD. We found that PR participants showed a high level of participation and completion, and clinically important improvements were seen in maximum exercise tolerance and quality of life.

At 85%, the PR completion rates in our study were high; other studies have shown that completion rates of programs of similar duration usually vary between 60% and 90%, but may be as low as 30%.^10^,^27^,^28^ The national report on PR in England and Wales involving >7,000 patients showed 71% of those enrolled completed the program.^29^ The high completion rates in the current study occurred despite profound transport problems in Kampala, and travel problems have been associated previously with higher dropout rates.^28^ While it was intended that participants would live within 10 km of the hospital, some participants came >50 km by bus twice a week and still attended regularly. This reflects an enthusiasm to take part seen in the majority of participants.

Care is needed in interpreting outcomes in a development study, which is not adequately statistically powered, and in a pre–post cohort design, causality cannot be implied. However, the changes associated with PR in outcomes such as disease-specific quality of life or exercise capacity were similar to, or larger than, the changes seen in conventional PR for COPD in Western countries. The main clinical outcomes assessed were the ISWT and the CCQ. The mean change in ISWT observed in this study of 79 m is well above the MCID of 48 m and greater than the difference between intervention and control groups of 40 m (range 22.4–57.1 m higher in eight studies) reported in a Cochrane review.^30^ Similarly for the CCQ, the magnitude of the changes seen in this development study is comparable or higher than other published data such as routinely collected data from a London teaching hospital program.^31^ Our data versus those from London were as follows: CCQ total −0.95 versus −0.5 and CCQ domain scores: symptoms −0.93 versus −0.1, mental state −1.22 versus 0.1, and CCQ functional −0.83 versus −0.2.

This study was conducted in patients with p-TBLD in Uganda, and there are many differences in such patients compared to patients with COPD in the Western World. They have different pathology, socioeconomic status, and limited access to conventional drug treatments. The desire to take part in the study was strong as there was little else on offer for them and the service was free. Many expressed gratitude, and this may have influenced the results of self-reported questionnaires. The participants in this program had TB-related lung damage, but many also had exposure to air pollution and tobacco smoke and nearly half had obstructive spirometry. Many were unable to work, had poor nutritional status, and may have impaired defense against damage: we believe that there is a complex interaction between infection, air pollution, and deprivation in causing chronic disease. PR, by improving ability to work and gain of weight and strength, may reverse the vicious circles and substantially improve health status.

This study confirmed the novel finding of the preceding, unpublished feasibility study that participants with p-TBLD noted reductions in chest pain after PR. Chest pain in p-TBLD is not widely reported, but it is present in 20% in a series of p-TBLD adult patients in Rwanda,^8^ and the rate was similar in an informal notes review we conducted in Mulago Hospital when designing the recruitment strategy. In this study, where participants had to have persistent breathlessness at baseline to be eligible to participate, 61% reported chest pain before and 25% 6 weeks after the end of PR. Of those with persistent pain after PR, mean pain severity scores were reduced in 57% of participants. The mechanism for this, and the less marked falls in hemoptysis, is not clear, and further adequately powered studies are needed to confirm the findings and examine underlying mechanisms.

Guidelines on TB treatment have largely omitted mention of the problem of p-TBLD, including the scale of the problem, how it is identified and assessed, and how the associated symptoms and disability can be improved. This study shows that PR is a potentially important treatment option for many people with p-TBLD, a finding that requires confirmation from larger controlled trials.

## Conclusion

p-TBLD is a widespread problem in many LMICs and affects people of all ages. There are no effective treatments currently available. Especially for the young, this is a huge problem in terms of disability, loss of income, and social isolation. PR addresses disability, exercise capacity, and social participation. This development study confirms that culturally appropriate PR in Uganda shows potential to improve a range of patient-related outcomes and health status and was associated with unexpected improvements in chest pains and hemoptysis. These findings need confirmation in adequately powered controlled trials.

## Figures and Tables

**Table 1:** Background characteristics of p-TBLD patients (n=29)

Characteristics	Value
Gender – female, n (%)	14 (51.7)
Age group (years), n (%)	
17–35	6 (20.7)
36–50	13 (44.8)
51–69	10 (34.5)
Age in years, mean (SD)	45 (13)
Highest level education, n (%)	
None/incomplete primary	11 (37.9)
Complete primary/incomplete secondary	9 (31)
Complete secondary/tertiary	9 (31)
Occupation, n (%)	
Unemployed	9 (31)
Others	20 (69)
Ever smoked, n (%)	10 (34.5)
If smoked, duration smoked in years, median (IQR)	15.5 (18)
Exposed to biomass, n (%)	12 (41.4)
If exposed to biomass	
Number of hours exposed per day, median (IQR)	2 (2)
Number of years exposed, median (IQR)	10 (15)

**Abbreviations:** p-TBLD, post-tuberculosis lung disorder; IQR, interquartile range.

**Table 2:** Baseline spirometric data on 29 participants

Variables	Prebronchodilator, mean (SD)	Postbronchodilator, mean (SD)
FEV_1_ (L)	1.40 (0.56)	1.42 (0.54)
FEV_1_ %	56.8 (25.0)	58.1 (25.2)
FVC (L)	2.08 (0.60)	2.13 (0.61)
FVC%	70.4 (21.8)	72.0 (21.63)
FEV:FVC	0.68 (0.19)	0.67 (0.19)

**Abbreviations:** FEV_1_, forced expiratory volume in 1 second; FEV_1_%, forced expiratory volume in 1 second as a percentage of predicted; FVC, forced vital capacity; FVC%, forced vital capacity as a percentage of predicted.

**Table 3:** Mean (95% CI) of health status, exercise capacity, and biometric outcomes for 29 participants who completed data collection at all three time points

Outcome measures	Baseline	End of PR	Change from baseline to end of PR	6 weeks after end of PR	Change from baseline to 6 weeks after end of PR
CCQ total score	1.88 (1.59, 2.17)	0.93 (0.76, 1.10)	−0.95 (−1.18, −0.72)	0.79 (0.63, 0.96)	−1.09 (−1.34, −0.83)
CCQ symptom score	2.03 (1.69, 2.38)	1.10 (0.88, 1.33)	−0.93 (−1.23, −0.63)	0.95 (0.71, 1.19)	−1.08 (−1.39, −0.79)
CCQ mental state score	1.98 (1.49, 2.47)	0.76 (0.47, 1.04)	−1.22 (−1.56, −0.89)	0.52 (0.33, 0.70)	−1.46 (−1.96, −0.97)
CCQ functional state score	1.67 (1.40, 1.95)	0.84 (0.69, 1.00)	−0.83 (−1.08, −0.57)	0.77 (0.60, 0.93)	−0.90 (−1.16, −0.65)
PHQ-9 total score	3.24 (1.24, 5.24)	0.03 (−0.04, 0.11)	−3.21 (−5.10, −1.31)	0.07 (−0.07, 0.21)	−3.17 (−5.05, −1.29)
Karnofsky score	75.86 (73.70, 78.02)	89.66 (88.05, 91.26)	13.79 (12.00, 15.59)	90.34 (88.19, 92.50)	14.48 (11.8, 17.19)
ISWT (m)	312.41 (275.95, 348.88)	402.41 (357.74, 447.08)	90.00 (56.50, 123.50)	402.07 (353.84, 450.29)	89.66 (49.32, 129.99)
Borg score after ISWT	6.48 (5.68, 7.28)	4.36 (3.55, 5.17)	−2.12 (−2.98, −1.26)	4.52 (3.65, 5.39)	−1.96 (−2.93, −1.00)
Sit-to-stand time (seconds)	10.45 (9.29, 11.61)	7.90 (7.01, 8.78)	−2.55 (−3.69, −1.41)	7.03 (6.53, 7.54)	−3.42 (−4.46, −2.37)
BMI^a^ (kg/m^2^)	20.86 (18.92, 22.81)	21.49 (19.59, 23.40)	0.63 (0.23, 1.03)	21.77 (19.92, 23.61)	0.91 (0.42, 1.39)
Mid upper arm circumference (cm)	25.27 (23.83, 26.72)	25.33 (23.10, 27.56)	0.06 (−1.64, 1.75)	25.92 (24.46, 27.39)	0.65 (0.21, 1.10)

**Note:**

a Based on 28 patients.

**Abbreviations:** CI, confidence interval; PR, pulmonary rehabilitation; CCQ, Clinical COPD Questionnaire; PHQ-9, Patient Health Questionnaire-9; ISWT, Incremental Shuttle Walking Test; BMI, body mass index.

**Table 4:** Number (%) of participants reporting cough, chest pains, and hemoptysis in 29 p-TBLD participants who completed data collection at all three time-points

Symptoms	Summary measure	Number (%) at time		
		Baseline	End of PR	6 weeks after end of PR
Experience pain in chest?	Yes	13 (44.8)	9 (31)	7 (24.1)
Suffer from hemoptysis?	Yes	5 (17.2)	2 (6.9)	2 (6.9)
Have cough?	None	17 (58.6)	14 (48.3)	17 (58.6)
	Mild	8 (27.6)	14 (48.3)	10 (34.5)
	Moderate	4 (13.8)	1 (3.4)	2 (6.9)
	Severe	0	0	0

**Abbreviations:** p-TBLD, post-tuberculosis lung disorder; PR, pulmonary rehabilitation.
